# Implementation of a standardized handoff system (I-PASS) in a tertiary care pediatric hospital

**DOI:** 10.1590/1984-0462/2023/41/2022123

**Published:** 2023-03-13

**Authors:** Deydson Rennan Alves Soares, Dalma Rodrigues, Fabio Carmona

**Affiliations:** a Universidade de São Paulo, Ribeirão Preto, São Paulo, Brazil.

**Keywords:** Patient handoff, Quality of health care, Patient safety, Hospital administration, Transferência da responsabilidade pelo paciente, Qualidade da assistência à saúde, Segurança do paciente, Administração hospitalar

## Abstract

**Objective:**

The handoff is the act of transferring information and responsibility among healthcare providers, and it is critical for the patient safety and the quality of service. The aim of this study was to evaluate the implementation of a standardized medical handoff system [I-PASS (Illness severity, Patient summary, Action list, Situation awareness and contingency planning, Synthesis by receiver)] and assess the effect on the amount and quality of the information transmitted during medical handoffs in a pediatric ward.

**Methods:**

In a prospective intervention study, physicians (staff and residents) who work in 12- or 24-h shifts in the pediatric ward of a single tertiary care Brazilian hospital were eligible. Those who agreed to participate were trained in an online session (lecture plus simulation). Medical handoffs were recorded pre- and post-intervention (training) to compare the amount and quality of information transmitted in handoffs.

**Results:**

The handoff standardization significantly increased the number of relevant information delivered for 12 out of the 16 items assessed without increasing, in seconds, the handoff duration (45.9 vs. 48.0; p=0.349). The protocol training and the following discussion about communication resulted in greater focus and attention among participants during transfers, decreasing time spent with interruptions and communication unrelated to the patient (18 vs. 2.7%). Regarding the I-PASS elements, there was an increase in the number of action lists and contingency plans reported (31 vs. 81% and 16 vs. 73%, respectively; p<0.001 for both).

**Conclusion:**

Standardization brought greater efficiency and objectivity to handoffs. It increased the quantity and quality of the information transmitted while successfully drawing attention to the most important points.

## RESUMO

**Objetivo:**

A passagem de plantão, ato de transferir informações e responsabilidade entre os médicos, é um dos elementos-chave para a qualidade do serviço prestado e a segurança do paciente. Este estudo objetivou avaliar a implantação de um sistema padronizado de passagem de plantão (*Illness severity, Patient summary, Action list, Situation awareness and contingency planning, Synthesis by receiver* — I-PASS) e avaliar o efeito sobre a quantidade de informações transmitidas em passagens de plantão de uma enfermaria pediátrica.

**Métodos:**

Estudo prospectivo de intervenção da implementação de um sistema padronizado de passagem de plantão (I-PASS). Foram realizadas gravações das passagens de plantão em período pré- e pós-intervenção. O treinamento dos médicos que participam das escalas de plantão foi feito em módulo teórico-prático, com o auxílio de plataformas digitais.

**Resultados:**

A padronização da passagem de plantão resultou em aumento do número de informações relevantes, entregues em 12 dos 16 itens pesquisados, sem aumentar a duração em segundos da transferência (45,9 *vs.* 48,0; p=0,349). O treinamento do protocolo aliado à discussão sobre estratégias de comunicação implicou maior foco e atenção durante as passagens, reduzindo o tempo gasto com interrupções e comunicações que não se referiam ao paciente (18 *vs.* 2,7%). No que se refere aos elementos do I-PASS, houve acréscimo na citação de pendências (31 *vs.* 81%, p < 0,001) e plano de contingenciamento (16 *vs.* 73%, p < 0,001).

**Conclusão:**

A padronização da passagem de plantão trouxe maior eficiência ao processo no que se refere a número de informações transmitidas, objetividade na transferência e atenção aos pontos importantes.

## INTRODUCTION

Medical errors are defined as “the failure of a planned action to be completed as intended or the use of a wrong plan to achieve an aim” and sometimes their frequency exceeds the number of deaths attributable to other causes, such as motor-vehicle wrecks, breast cancer, and AIDS.^1^ The first studies addressing adverse events associated with healthcare were published by the end of the 1970s. Adverse events often result in longer hospital stays or disabilities.^1^ Given the relevance of the matter, the World Health Organization (WHO, 2005) proposed the key concepts and a definition for patient safety. It also suggested strategies to decrease risks and the incidence of healthcare-associated adverse events.^2^ Later on, the National Program for Patient Safety was created in Brazil in 2013.^3^

Communication failure is one of the leading causes of healthcare-associated adverse events, corresponding to up to 70% of the causes.^4^ In this context, the WHO considers patient handoffs, the act of transferring information and responsibility for patients between healthcare professionals, as a critical aspect of the quality of care. Patient handoffs are especially susceptible to miscommunication and are a potential source of error and adverse events, so strategies to improve the quality and clarity of relevant information being transferred are highly desirable.^5^ In 2014, an intervention study carried out in 11 pediatric academic centers in the United States and Canada implemented a standardized handoff system called I-PASS (Illness severity, Patient summary, Action list, Situation awareness and contingency planning, Synthesis by receiver), which resulted in a 23% decrease in medical errors and a 30% decrease in preventable adverse events in hospitalized children. Each letter in I-PASS corresponds to a block of relevant information for handoffs: I (Illness severity), which is the patient’s disease severity, requiring more or fewer resources, being stable, “watcher,” or unstable; P (Patient summary), which includes a summary statement containing the events leading up to admission, hospital course, ongoing assessment, and plan; A (Action list), which refers to a to-do list, along with timeline and ownership; S (Situation awareness and contingency planning), which brings attention to potential adverse events, complications, or clinical deterioration, plus contingency plans; and the second S (Synthesis by the receiver), which is the synthesis by the receptor, when they summarize what was heard, ask questions, and restate key actions or to-do items.^6,7^ Evidence shows that the implementation of I-PASS resulted in the inclusion of key information elements in patient handoffs without increasing handoff duration.^8,9^

At HC Criança, the pediatric unit at the Hospital das Clínicas da Faculdade de Medicina de Ribeirão Preto, Universidade de São Paulo (HCFMRP-USP), there is no standardized handoff system, nor reliable statistics on medical errors or preventable adverse events. Thus, we aimed to implement I-PASS at HC Criança and assess whether staff training on I-PASS would result in better quality handoffs with more frequent inclusion of the I-PASS elements.

## METHOD

This was a before-after intervention study. It was designed according to the SPIRIT (Standard Protocol Items: Recommendations for Interventional Trials) and SQUIRE (Standards for Quality Improvement Reporting Excellence) recommendations.^10,11^ The study was approved by our local Research Ethics Committee (CAAE: 32073520.3.0000.5440), and a signed informed consent was required from all physicians. The Research Ethics Committee waived the need for signed consent forms for patients.

The study was conducted in the pediatric ward at HC Criança. In this tertiary care ward, 12-h nightshifts run on weekdays, while 24-h shifts run on weekends and holidays. The shift teams include a senior hospitalist physician (staff), a second-year pediatric resident, and a first- or second-year resident in a pediatric specialty. Handoffs are verbal but aided by an online spreadsheet containing semi-structured patient summaries (Google Docs^®^, Google Alphabet, Mountain View, CA, EUA).

Participation was voluntary. All physicians (residents and staff) taking shifts during the study were eligible. The inclusion criteria were being on shifts during the study and signing the informed consent. The exclusion criterion was the participant’s request. In total, 33 physicians participated in pre-intervention data collection (10 second-year pediatric residents, 15 first- or second-year residents in a pediatric specialty, and 8 senior hospitalist physicians). Regarding the post-intervention collection, 23 physicians participated (5 second-year pediatric residents, 12 first- or second-year residents in a pediatric specialty, and 6 senior hospitalist physicians). There was no repetition of the second-year pediatric residents, as there was a change in rotation during the collections. Regarding the resident physicians in the pediatric practice area and physician-hospitalists, there was a repetition of 11 residents and 6 physician-hospitalists.

I-PASS materials are available upon request at their webpage (http://www.ipasshandoffstudy.com) and were translated to Brazilian Portuguese by an experienced English-Portuguese translator (DR). Residents and staff taking shifts in the pediatric ward and who agreed to participate were trained by a research team member (DRAS) in an online session (lecture plus simulation), as described below: an explanation of the rationale and goals of I-PASS, followed by a 2-h workshop on teamwork and communication skills and a 1-h simulation session with clinical handoff scenarios, including feedback from participants. All the activities were performed on the same day with a total duration of 210 min. Online training was mandatory due to the restrictions imposed by the COVID-19 pandemic in 2020. Therefore, an online course was created in the Moodle platform^12^ from the University of São Paulo (cursosextensao.usp.br) with reading and video assignments and assessment tools. Live simulation sessions were held in small groups (up to three) via web conference (Google Meet^®^, Google Alphabet, Mountain View, CA, EUA).

Assuming a 50% frequency of I-PASS elements before intervention and a relative increase of 30% in this frequency, significance of 5%, and power of 80%, handoffs of 167 patients would be needed in each phase.

Data were tabulated in a Microsoft Excel^®^ spreadsheet (Microsoft Corporation, Redmond, WA, EUA). Descriptive statistics were expressed as absolute and relative frequencies, means (standard deviations), or medians (interquartile ranges), when appropriate. Pre- and post-intervention frequencies of I-PASS elements were compared with a chi-square test. The mean time spent with each patient during pre- and post-intervention handoffs was compared with a Student’s t-test. A significance level of 5% was adopted.

## RESULTS

Pre-intervention recordings of 29 handoffs for 720 patients were done between August 5 and September 23, 2020, summing up 11 h, 15 min, and 20 s of audio (23.3 min per handoff on average). About 18% (2 h) of the audio was unrelated to the patients (irrelevant comments and interruptions). The average time spent for each patient was 45.9 s. In this phase, patients were primarily male (63.3%), aged between 1 month and 16 years, but mostly younger than 1 year. The interval between collections was approximately 4 months (114 days).

Post-intervention recordings of 20 handoffs for 549 patients were done between January 15 and February 28, 2021, totaling 7 h, 31 min, and 50 s of audio (22.6 min per handoff on average). The proportion of time spent with unrelated comments and interruptions was only 2.7% (about 12 min). In this phase, patients were primarily male (56.5%), and their ages varied from 1 month to 18 years, most being younger than 1 year. The average time spent for each patient was 48.0 s, not significantly different from the pre-intervention (vs. 45.9 s, p=0.349).

The distribution of patients according to the pediatric specialty they were admitted to, along with their severity, is presented in Table 1. Pre- and post-intervention, most patients were seen by gastroenterology and oncology and were stable. For each category of patient severity, the median duration of individual handoffs is shown in Table 2. In short, unstable patients took longer to handoff than intermediate and stable patients, as expected, while the intervention did not affect handoff duration.

**Table 1.** Distribution of patients according to the pediatric specialty they were admitted to and to their severity, pre-intervention and post-intervention.

	Pre-intervention (n=720)	Post-intervention (n=549)
Pediatric specialty (%)		
Cardiology	78 (10.8)	48 (8.7)
Pediatric surgery	41 (5.7)	21 (3.8)
Endocrinology	62 (8.6)	42 (7.7)
Gastroenterology	204 (28.3)	131 (23.9)
Nephrology	65 (9.0)	21 (3.8)
Neurology	88 (12.2)	43 (7.8)
Oncology	94 (13.1)	132 (24.0)
Pneumology	24 (3.3)	51 (9.3)
Rheumatology	33 (4.6)	31 (5.7)
Other	31 (4.3)	29 (5.3)
Severity (%)		
Stable	597 (82.9)	484 (88.2)
Intermediate	115 (16.0)	63 (11.5)
Unstable	8 (1.1)	2 (0.4)

Values are expressed as absolute (relative) frequencies.

**Table 2.** Duration of handoffs (seconds) per patient recorded pre- and post-intervention, according to patient severity.

Patient severity	Pre-intervention (n=720)	Post-intervention (n=549)
Stable	30 (20, 45)	40 (25, 50)
Intermediate	70 (45, 105)	85 (65, 110)
Unstable	138 (69, 274)	145*

Values are expressed as median (interquartile range); *interquartile range is not calculated due to few observations in this category.

Regarding the essential elements in handoffs, Table 3 presents the number of mentions of each item (in frequencies) on pre- and post-intervention periods. There was an increase in the number of mentions for 12 out of the 16 items assessed.

**Table 3.** Frequencies of mentions to essential elements during handoffs recorded pre- and post-intervention.

Elements	Pre-intervention (n=720) (%)	Post-intervention (n=549) (%)
Name	720 (100.0)	549 (100.0)
Age	299 (41.5)	460 (83.8)
Gender	720 (100.0)	549 (100.0)
Admission reason	573 (79.6)	549 (100.0)
Patient summary	526 (73.1)	549 (100.0)
Past medical history	247 (34.3)	398 (72.5)
Allergies	4 (0.6)	1 (0.2)
Weight	4 (0.6)	8 (1.5)
Physical examination	268 (37.2)	311 (56.7)
Devices	158 (21.9)	137 (25.0)
Lab tests and imaging	275 (38.2)	234 (42.6)
Vital signs	140 (19.4)	166 (30.2)
Feeds and medications	421 (58.5)	448 (81.6)
To do list	225 (31.3)	446 (81.2)
Contingency plan	115 (16.0)	400 (72.9)
Advanced directives of will	12 (1.7)	7 (1.3)

Values are expressed as absolute (relative) frequencies. I: Illness severity; P: Patient summary; A: Action list; first S: Situation awareness and contingency planning; second S: Synthesis by the receiver; *p<0.001 by chi-square test.

**Figure 1.**
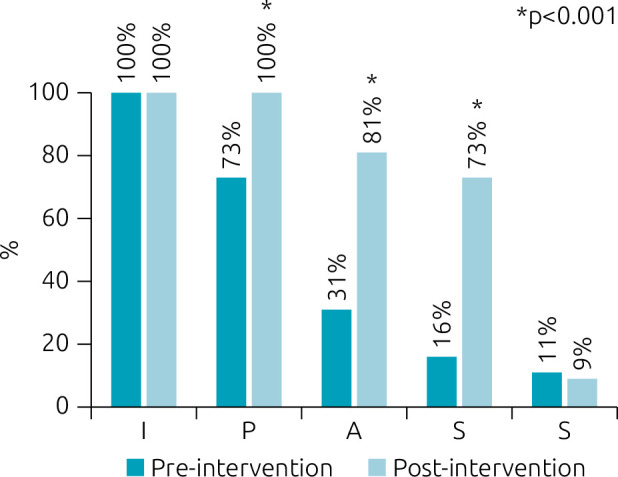
Relative frequencies of mentions to the I-PASS elements in handoffs before and after the intervention.

Analyzing I-PASS elements, we found that illness severity (I) was always mentioned, while patient summary (P) was cited for 73% of patients during pre-intervention. This number increased to 100% post-intervention. Action list (A), situation awareness, and contingency plan (S) were much less mentioned before the intervention, but their mention increased significantly after the intervention (both p<0.001). Lastly, synthesis by the receiver (S) was seldom mentioned before the intervention, and this number did not increase after the intervention (Figure 1).

I: Illness severity; P: Patient summary; A: Action list; first S: Situation awareness and contingency planning; second S: Synthesis by the receiver; *p<0.001 by chi-square test

## DISCUSSION

We showed that adopting I-PASS, a standardized protocol for handoffs, in a pediatric ward resulted in a significant increase in the amount and quality of information transmitted during handoffs without increasing their duration.

We speculate that the effect we observed was not only due to the training of physicians on the I-PASS mnemonic but also because of the discussions about teamwork, effective communication, and preventable adverse events and errors. Caring for patients during hospital admission requires a set of skills that includes teamwork and effective communication. These skills have been more appreciated and valued in the past decades. Students in the health sciences need to be appropriately trained and stimulated to develop these skills further since most adverse events and errors happen as a direct or indirect consequence of poor communication.^13^ We believe these discussions augmented their focus and attention during handouts since there was also a decrease in the time spent with interruptions or other topics unrelated to the patients.

Previous studies have reported experiences of different hospitals with standardized handoffs. In 2014, in the United States, Starmer et al. showed that, after I-PASS implementation, the medical error rate decreased by 23%, and the rate of preventable adverse events decreased by 30% without increasing the duration of handoffs or resident workload. In our study, the duration of handoffs was not increased as well.^7^ Keebler et al. conducted a meta-analysis to investigate whether standardized handoff protocols would increase handoff information passed during transitions of care, patient outcomes, provider outcomes, and organizational outcomes. They found positive effects on all outcomes, but publication bias and methodological issues weakened the conclusions.^14^ Another example is the study by Nedved et al., in the United States, in 2021. In their study, healthcare providers reported positive perceptions of the overall quality of communication without changes in the mean duration of handoffs.^15^

In a systematic review, Desmedt et al. found that poor handoff is associated with several potential hazards to patients but also point out that there is no single best tool or high-quality evidence of what constitutes best handoff practices. Nevertheless, they highlighted that standardized tools do facilitate coordination of care and communication.^16^

While most handoff elements were more frequently mentioned after the intervention, some were not. Regarding the elements fewer mentioned, as allergies and advanced directives of will, we attributed the low number of mentions to the fact that they were absent. Weight was also rarely mentioned, only if highly abnormal, probably because this information is always present in the online spreadsheet that serves as a patient summary. It is noteworthy that three I-PASS elements were more frequently mentioned after the intervention [patient summary (P), action list (A), and situation awareness/contingency plan (S)]. We believe this fact was accompanied by better clinical judgment and problem anticipation, which may have benefited our patients.

Surprisingly, the frequency of the item synthesis by the receptor (S) decreased by 12%. We believe our physicians did not adhere to this item because they thought they were wasting time repeating information already shared. We also believe this is a cultural aspect of our service since a similar study showed increases in mentions of all I-PASS elements (to 97–100%) in the United States.^17^ Maybe our physicians need more training to reinforce the importance of bidirectional communication in teamwork.

In fact, continuous or repeated training is needed. A recent study by Tufts et al. assessed the inclusion of I-PASS elements in handoffs a couple of months after training. They found that adherence to I-PASS was not sustained and suggested continuous training of residents and staff to maintain the quality of handoffs.^18^ Desmedt et al. stated, in their systematic review, that teaching handoff methods such as I-PASS with role-playing and simulation may result in better learning and incorporation into practice and, therefore, better healthcare.^16^ In addition to the technical-operational conditions related to in-service training and the environment’s infrastructure, it is important to mention that there is a need for a change in the motivational culture of professionals aimed at understanding the structuring of the shift change as something intrinsic to patient safety.

Besides the lack of standardization, another critical factor that may lead to a poor handoff is the environment. A noisy room with people coming in and out and frequent interruptions may substantially impair the quality of information being transmitted. In our study, the time spent with interruptions or matters unrelated to the patients was significantly shorter after the intervention. Since we did not change the room where handoffs took place, we speculate that physicians were more focused on communication after training. Other measures that can be implemented in the future include a more reserved, quiet room for handoffs and a door sign with “Do not interrupt unless it’s an emergency,” among others.

Our study has several limitations. First, on some days, handoffs of some patients were done separately because some staff was not available at the time of the general handoff. This mainly occurred in the pre-intervention period, so we increased the number of recordings to accommodate for this and avoid sampling bias. Second, for administrative reasons, we could not assess the effect of adopting I-PASS on the rates of errors and preventable adverse events. Although the impact of I-PASS on these events is already shown in the literature in other countries, we could not replicate these results in our setting. This needs to be addressed in the future. Third, the number of physicians trained was relatively small since the study was done in a single pediatric ward. We plan to expand training hospital-wide. In addition, the absence of blinding in relation to the time of collection may have led to a better performance of the second collection team in relation to the first.

Training physicians and adopting the I-PASS protocol for handoffs in a single tertiary care pediatric ward increased the quantity and quality of information transmitted between shifts and decreased the relative time spent with interruptions and other subjects without increasing the mean handoff duration.
